# Comparative Analysis of Bond Strength in Endodontic Access: Immediate Endodontic Sealing Versus Delayed Endodontic Sealing

**DOI:** 10.7759/cureus.115871

**Published:** 2026-09-06

**Authors:** Thanyaporn Buranakarn, Yanee Tantilertanant, Junji Tagami, Somsinee Pimkhaokham

**Affiliations:** 1 Department of Operative Dentistry, Faculty of Dentistry, Chulalongkorn University, Bangkok, THA; 2 Department of Cariology and Operative Dentistry, Graduate School of Medical and Dental Sciences, Institute of Science Tokyo, Tokyo, JPN

**Keywords:** adhesive dentistry, endodontic access, etch-and-rinse adhesives, immediate pre-endodontic sealing, optibond fl, single bond universal adhesive, sodium hypochlorite

## Abstract

Introduction: Coronal leakage and restoration failure remain significant contributors to the failure of endodontic treatments. This study aimed to evaluate the influence of different adhesive systems and treatment protocols, specifically immediate endodontic sealing (IES) and delayed endodontic sealing (DES), on bond strength.

Materials and methods: Sixty extracted human mandibular third molars were prepared to expose the pulp chamber dentin and randomly assigned to six groups according to the adhesive systems (OptiBond™ FL (Kerr Dental, California, United States) or Single Bond Universal adhesive (Solventum Corporation, Eagan, Minnesota, United States)) and the treatment protocols (control, IES, and DES). Sodium hypochlorite irrigation was performed based on experimental protocols. Following composite restoration, specimens were thermocycled for artificial aging and subsequently sectioned into beams for microtensile bond strength (µTBS) testing (10 teeth per group). Data were analyzed using two-way ANOVA and Kruskal-Wallis with Dunn's post hoc test (α = 0.05).

Results: A significant interaction between adhesive system and treatment protocol was identified (p < 0.05). With OptiBond FL, IES produced significantly higher µTBS values than DES (52.85 ± 7.22 MPa vs 40.98 ± 5.76 MPa; p < 0.05) and was comparable to the control group (57.59 ± 7.18 MPa; p > 0.05). The OptiBond FL group demonstrated significantly higher bond strength than the Single Bond Universal group under all conditions (p < 0.05), whereas the IES-Single Bond Universal adhesive (34.43 ± 5.97 MPa) and DES-Single Bond Universal adhesive (30.27 ± 5.49 MPa) groups showed significantly lower µTBS values than the control-Single Bond Universal adhesive (41.12 ± 4.85 MPa; p < 0.05). Failure mode analysis revealed predominantly adhesive failures in all groups.

Conclusions: Within the limitations of the in vitro study, IES with OptiBond FL resulted in higher µTBS than DES.

## Introduction

The success of endodontically treated teeth (ETT) is strongly influenced by the quality and long-term durability of the coronal restoration. Clinical evidence indicates that restorative failure is the primary cause of clinical complications in these cases [1]. These failures underscore the necessity of good-quality bonding of the restorative material to tooth structure, which is vital for maintaining an effective coronal seal and increasing the tooth's resistance to fracture [2]. As minimally invasive restorative and conservative access opening concepts allow ETT to be restored using direct restorations [3] or partial bonded restorations, including inlays and onlays [4], reliable adhesive bonding becomes fundamental to clinical success.

Achieving this bond, however, is complicated by disinfection protocols. While freshly cut dentin has been reported to offer high bonding potential, sodium hypochlorite (NaOCl), a commonly used endodontic irrigant, may adversely affect the dentin substrate. NaOCl has been reported to compromise the quality of the hybrid layer through collagen degradation [5] and the release of oxidizing by-products that may interfere with resin polymerization [6]. Collectively, these alterations may impair the performance of adhesive restorations following endodontic treatment.

Rather than replacing this essential chemical irrigation protocol, a strategic alternative treatment protocol has recently been introduced to mitigate these adverse effects without compromising disinfection efficacy. Immediate endodontic sealing (IES), adapted from the immediate dentin sealing (IDS) concept used in restorative dentistry [7], involves the application of an adhesive resin to the access cavity immediately after access cavity preparation and before root canal irrigation. This approach aims to shield freshly cut dentin from chemical contamination and collagen degradation throughout the endodontic procedure. Previous studies have demonstrated that IES enhances the internal adaptation of resin composite to dentin [8] and provides significantly higher fracture resistance compared with delayed endodontic sealing (DES) [9].

To maximize the benefits of this strategy, multi-step etch-and-rinse adhesive systems have been recommended for IDS [7,10], providing a rationale for their application in the IES approach. However, the development of adhesive systems has shifted toward the simplification of techniques like universal adhesives. These systems are claimed to simplify clinical procedures and reduce technique sensitivity through their multi-mode versatility. Despite these claims, limited evidence exists regarding the effectiveness and long-term performance of universal adhesive systems when utilized specifically within the IES approach [11].

This raised the question of whether IES with universal adhesive minimized the adverse effects of endodontic irrigation on bonding efficacy. Therefore, the objective of this study was to evaluate the effect of IES compared to DES on the microtensile bond strength (μTBS) of coronal restorations to pulp chamber dentin using multi-step etch-and-rinse adhesive and universal adhesive. The null hypotheses were: (i) there was no significant difference in μTBS to dentin in the pulp chamber between IES and DES treatment protocols, and (ii) there was no significant difference in μTBS to pulp chamber dentin between the two types of adhesive systems used in IES and DES treatment protocols.

## Materials and methods

This was an in vitro study conducted at the Department of Operative Dentistry, Faculty of Dentistry, Chulalongkorn University, Bangkok, Thailand. The research proposal was approved by the Human Research Ethics Committee of the Faculty of Dentistry, Chulalongkorn University (study code: HREC-DCU 2025-011, dated March 7, 2025). Informed consent was obtained from all patients from whom teeth were extracted. For patients under 18 years of age, informed consent was obtained from a parent or legal guardian.

Inclusion criteria

The inclusion criteria included mandibular molars with a cemento-enamel junction (CEJ) circumference of 32 ± 1.5 mm and a circumference of 37 ± 1.5 mm at a level 4 mm coronal to the CEJ. The internal dimensions of the selected teeth were assessed radiographically using a digital intraoral sensor (70 kV, 7 mA, 0.183 s) (Nanopix; Changzhou Sifary Medical Technology Co., Ltd., Changzhou, Jiangsu, China) in an occlusal view. A minimum residual dentine thickness of 2.5 mm was confirmed in both the buccolingual and mesiodistal dimensions.

Sample size calculation and group segregation

An a priori sample size calculation was performed using a software program (G*Power 3.1.9.4; Heinrich-Heine University Düsseldorf, Germany) with an F test for a one-way ANOVA (fixed effects, omnibus) across six experimental groups representing the combinations of two adhesive systems, OptiBond™ FL (OFL) (Kerr Dental, California, United States) and Single Bond Universal adhesive (SBU) (Solventum Corporation, Eagan, Minnesota, United States), and three treatment protocols, control, IES, and DES. The effect size was derived from μTBS values of two adhesive systems reported in a previous study [12], yielding an effect size of f = 0.527. With α = 0.05 and power = 0.80, the minimum required total sample size was 54 specimens. This was increased by approximately 10% to 60 specimens to compensate for potential specimen loss during preparation and testing.

The 60 teeth included for μTBS testing were divided into six groups with 10 teeth in each group. Specimens were stratified by tooth size and, within each stratum, randomly allocated to one of six experimental groups using a random number sequence generated in Microsoft Excel (RAND function) (Microsoft Corporation, Redmond, Washington, United States). Allocation was not concealed from the operator because the same investigator generated the sequence and performed the experimental procedures.

Sample collection and storage

A total of 60 sound human third molars were extracted from patients aged 16-30 years following informed consent. Blood and adherent tissues were gently removed using an ultrasonic scaler (Guilin Woodpecker Medical Instrument Co., Ltd., Guilin, Guangxi, China) under running water. Teeth were stored in a 0.5% aqueous solution of Chloramine-T for at least seven days at room temperature, followed by storage in distilled water. All teeth were used within six months after extraction. Before experiments, all teeth were examined under a stereomicroscope (SZ 61; Olympus Corporation, Hachioji, Tokyo, Japan) for visible cracks, caries, restorations, or other defects, which were excluded.

Specimen preparation

**Figure 1:**
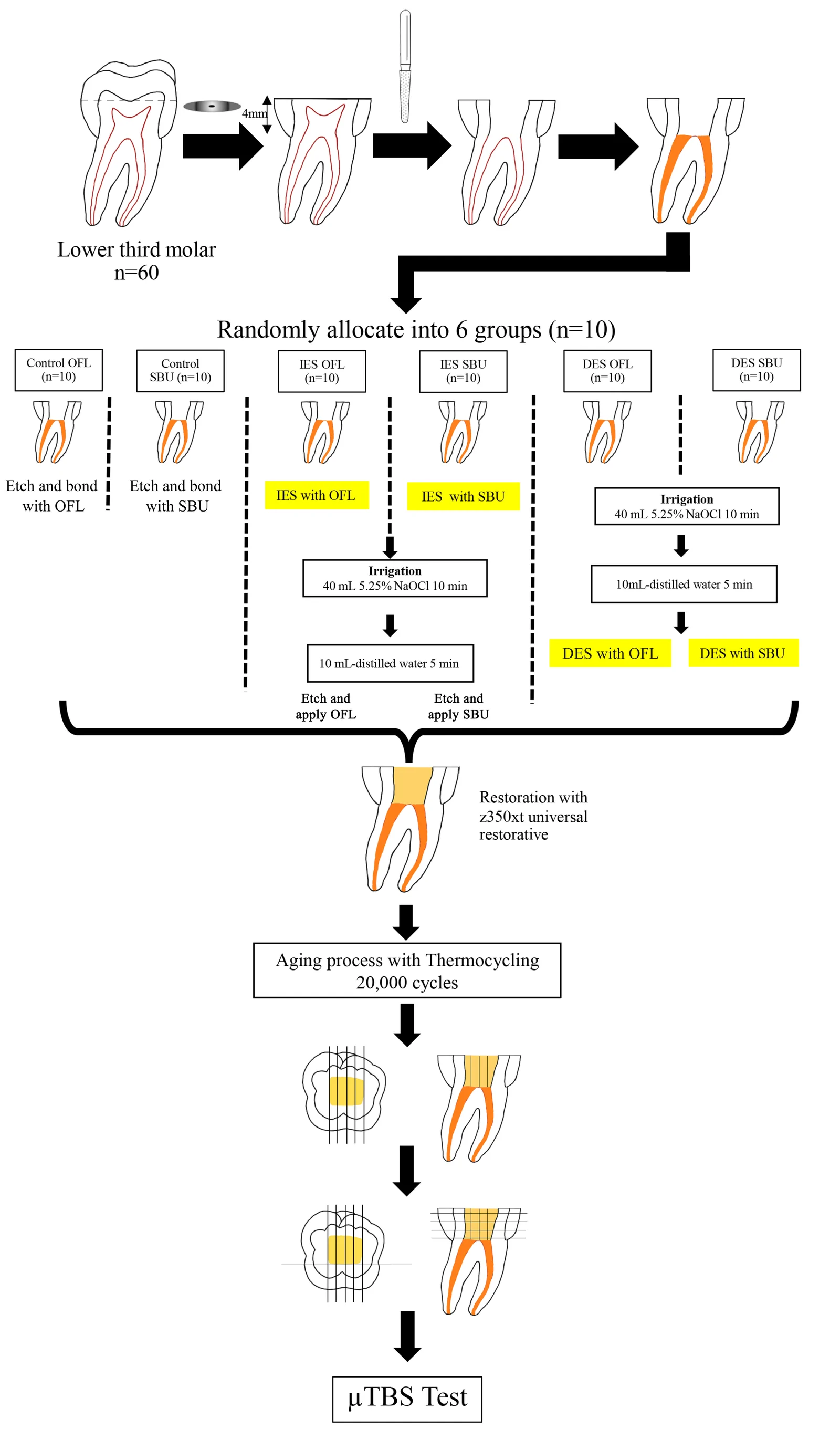
Diagrammatic presentation of experiment protocol Specimens were prepared by exposing the pulp chamber while preserving 4 mm of remaining dentin from the occlusal surface to the pulpal floor. The root canal orifices were sealed with gutta-percha before restoration. Teeth were restored according to the six experimental protocols and subjected to 20,000 thermocycles. Subsequently, specimens were sectioned to obtain approximately 1.0 ± 0.1 mm beam-shaped specimens for μTBS testing. OFL: OptiBond FL (Kerr Dental, California, United States); SBU: Single Bond Universal (Solventum Corporation, Eagan, Minnesota, United States); IES: immediate endodontic sealing; DES: delayed endodontic sealing; μTBS: microtensile bond strength Image Credit: Thanyaporn Buranakarn; created using Adobe Photoshop 2026 (Adobe Inc., San Jose, California, United States)

The occlusal portion of each tooth was sectioned to obtain 4 mm of remaining tooth depth from the occlusal surface to the pulpal floor with a slow-speed, water-cooled diamond saw (IsoMet™ 1000 Precision Cutter; Buehler, Lake Bluff, Illinois, United States). The remaining roof was then removed using a tapered round-end diamond bur (FG D8; Intensiv SA, Collina d'Oro, Switzerland) operated in a high-speed handpiece under water cooling to fully expose the pulp chamber. Each bur was replaced after five preparations to maintain cutting efficiency. The pulp tissue was removed using the Maillefer Barbed Broach (Dentsply Sirona Inc., Charlotte, North Carolina, United States). The root canal orifices were then covered with gutta-percha to prevent restorative material from entering the canal space. The materials used in this study are listed in Table 1. The experimental grouping and the sequence of bonding, irrigation, and restorative procedures are shown in Figure 1.

**Table 1:** Composition of adhesive systems, etching agents, and resin composite Bis-GMA: bisphenol A diglycidyl ether dimethacrylate; HEMA: 2-hydroxyethyl methacrylate; TEGDMA: triethylene glycol dimethacrylate; 10-MDP: 10-methacryloyloxydecyl dihydrogen phosphate; UDMA: urethane dimethacrylate; GPDM: glycerol phosphate dimethacrylate; PAMM: methacryloxy ethyl phthalate; Bis-EMA: ethoxylated bisphenol A glycol diamethacrylate

Material	Main component	pH	Manufacturer
OptiBond^TM^ FL	Primer: HEMA, GPDM, PAMM, ethanol, water, photoinitiator	1.8	Kerr Dental, California, United States
	Adhesive: TEGDMA, UDMA, GPDM, HEMA, Bis-GMA, filler, photoinitiator	6.9	Kerr Dental, California, United States
Single Bond^TM^ Universal Adhesive	Adhesive: 10-MDP, Vitrebond copolymer, HEMA, dimethacrylate resins, filler, silane, initiator, ethanol, water	2.7	Solventum Corporation, Eagan, Minnesota, United States
Kerr OptiBond^TM^ Gel Etchant	37.5% Phosphoric acid, silica thickener	0.5-1.5	Kerr Dental, California, United States
Scotchbond^TM^ Universal Etchant	32% Phosphoric acid, polyvinyl alcohol	0.1	Solventum Corporation, Eagan, Minnesota, United States
Filtek^TM^ Z350XT Universal Restorative	Inorganic phase: Silica (20 nm non-agglomerated aggregated), zirconia (4-11 nm non-agglomerated/aggregated and TEGDMA agglomerated), clusters, zirconia/silica aggregated particles (20 nm silica particles combined with 4-11 nm zirconia)	N/A	Solventum Corporation, Eagan, Minnesota, United States
	Organic phase: Bis-GMA, UDMA, TEGDMA, Bis-EMA		

Irrigation and bonding protocol

After tooth preparation, the irrigation and bonding protocols were conducted according to assigned groups as follows. The control groups of both adhesive systems, control-OFL and control-SBU, received no irrigation. The composite restoration was placed immediately after adhesive application. In the IES groups of both adhesive systems (IES-OFL and IES-SBU), the bonding procedure was conducted before the irrigation procedure. After NaOCl irrigation, the complete bonding procedure was redone according to the assigned adhesive system, as described in Table 2. This included re-etching with the designated phosphoric acid etchant followed by re-application of both the primer and adhesive for IES-OFL and re-application of SBU for IES-SBU. No mechanical surface refreshment was performed before the second bonding procedure. Then, the resin composite restoration was performed. Meanwhile, in the DES groups (DES-OFL and DES-SBU), irrigation was performed prior to the bonding procedures, followed by composite restoration.

**Table 2:** Technical procedures for each adhesive system and resin composite used in the study

Materials	Application steps recommended by manufacturer's instructions
OptiBond^TM^ FL (Kerr Dental, California, United States)	Etch: Apply OptiBond Gel Etchant (Kerr Dental, California, United States) for 15 seconds, rinse with water for 15 seconds, and gently air dry to achieve moist dentin
	Prime: Apply primer with a light scrubbing motion for 15 seconds, and gently air dry for 5 seconds
	Bond: Apply a thin coat of adhesive, rub off the excess adhesive, and light cure for 20 seconds
Single Bond^TM^ Universal Adhesive (Solventum Corporation, Eagan, Minnesota, United States)	Etch: Apply Scotchbond Universal Etchant (Solventum Corporation) for 15 seconds, rinse with water for 15 seconds, and gently air dry to achieve moist dentin
	Bond: Apply adhesive and rub for 15 seconds, rub off the excess adhesive, and light cure for 10 seconds
Filtek Z350XT Universal Restorative (Solventum Corporation, Eagan, Minnesota, United States)	Apply in 2 mm increments and light cure 40 seconds for each layer

The bonding protocol in each group was conducted following the manufacturer’s instructions. The application procedures of all materials used in this study are presented in Table 2. For the irrigation protocol, the tooth was irrigated with 40 mL of 5.25% NaOCl [13] at a flow rate of 4 mL/minute [14] using a conventional needle syringe, followed by a final rinse with distilled water for 10 mL to reduce the amount of residual NaOCl and limit its continued action.

Restoration protocol

All groups were restored with a nanofilled resin composite (Filtek Z350XT Universal Restorative; Solventum Corporation). After this, the resin composite was cured with a light-emitting diode (LED) light-curing unit (Demi™ Plus Curing Light; Kerr Dental). The light-curing unit was placed perpendicular to the occlusal surface of the tooth for 40 seconds. For every six specimens, the light-curing unit was calibrated with an Optilux radiometer (Kerr Dental).

Artificial aging protocol

After restoration, specimens were stored in water at 37 °C for one day before artificial aging with 20,000 thermocycles (THE1400 Thermocycler; SD Mechatronik GmbH, Feldkirchen-Westerham, Germany) in distilled water with temperatures of 5 °C and 55 °C, a 5-second transfer, and a 30-second dwell time, as an artificial-aging protocol.

Tooth sectioning for μTBS testing

All thermocycled teeth were sectioned with the IsoMet 1000 Precision Cutter using the non-trimming technique into approximately 1 × 1 mm beams [15]. Each tooth was then vertically sectioned in the mesiodistal direction into 1.0 ± 0.1 mm slices down to the pulpal floor, and the lingual segment was removed by an additional vertical cut to obtain the buccal segment. Horizontal sectioning in the buccolingual direction was subsequently performed to obtain approximately 1.0 ± 0.1 mm thick beam-shaped specimens. At least six beams were produced per tooth. The fractured or defective beams were excluded. Beams that fractured before μTBS testing were classified as pre-test failures, whereas beams with incomplete cross-sectional areas or dimensions that did not meet the specified 1 × 1 mm² criteria were classified as defective. The number of pre-test failures per tooth and per experimental group is presented in Table 3. To confirm that pre-test failure rate did not differ meaningfully between groups, a chi-square test was performed (χ² = 1.27, df = 5, p = 0.938); given expected cell counts below 5, Fisher's exact test was also applied and yielded a comparable result (p = 0.933). Both tests indicate no significant association between experimental group and pre-test failure rate. Each tooth was considered as an experimental unit (n = 10 teeth per group), and the mean μTBS value for each tooth was calculated from all successfully tested beams obtained from that tooth and used for statistical analysis.

**Table 3:** The number of completed, pre-test failure, and defective beams per tooth and experimental group IES: immediate endodontic sealing; DES: delayed endodontic sealing; OFL: OptiBond FL (Kerr Dental, California, United States); SBU: Single Bond Universal adhesive (Solventum Corporation, Eagan, Minnesota, United States); C: completed beam; F: pre-test failure beam; D: defective beam

Tooth	Control-OFL			IES-OFL			DES-OFL			Control-SBU			IES-SBU			DES-SBU		
	C	F	D	C	F	D	C	F	D	C	F	D	C	F	D	C	F	D
1	8	2	0	6	2	0	8	0	0	6	0	0	8	0	0	7	1	0
2	8	0	0	6	0	2	6	0	1	8	0	0	8	0	0	6	1	0
3	6	0	0	8	0	0	7	0	0	6	0	1	8	1	0	6	0	0
4	7	0	0	8	0	0	8	0	0	6	0	1	7	0	0	6	0	0
5	7	0	0	6	1	0	7	0	0	6	0	0	7	0	0	6	0	0
6	7	0	0	7	0	0	6	0	1	6	1	0	7	1	0	6	0	0
7	8	0	0	8	0	0	7	1	0	6	0	0	7	0	0	6	2	1
8	6	0	0	6	0	0	6	1	0	6	1	0	6	0	0	6	0	0
9	6	0	0	7	0	0	6	1	0	6	0	0	6	0	1	8	0	0
10	6	0	0	6	0	0	6	0	0	6	1	0	6	0	0	6	0	1
Total	69	2	0	68	3	2	67	3	2	62	3	2	70	2	1	63	4	2

μTBS testing

The cross-sectional area of each beam was measured using a digital caliper (CD-6" CS; Mitutoyo Corporation, Kawasaki, Kanagawa, Japan) before testing. Each specimen was mounted to a universal testing machine (EZ-S; Shimadzu Corporation, Nakagyo-ku, Kyoto, Japan) using cyanoacrylate adhesive (Dentsply Sirona). μTBS testing was subsequently performed with a crosshead speed set at 1 mm/minute under a 500-N load cell. All beams obtained from each group were subjected to µTBS testing. The µTBS of each beam was calculated and expressed in megapascals (MPa).

Failure mode analysis

After μTBS testing, the fractured surface of both dentin and composite sides was evaluated by a stereomicroscope (SZ 61; Olympus Corporation) at 45x magnification and classified as follows: adhesive failure was defined as fracture at the interface between dentin and adhesive or at the interface between adhesive and resin composite, or within the adhesive layer. Cohesive failure in composite was defined as a fracture within the resin composite, and cohesive failure in dentin was defined as a fracture in dentin. Mixed failure was defined as the presence of both adhesive and cohesive failure components within the same specimen, regardless of the extent of each component. The recorded numbers for each mode were calculated across all fractured beams within each group and presented as percentages.

Scanning electron microscope (SEM) analysis

Two randomly selected representative fractured ends from each group were further analyzed under a SEM (JEOL JSM‑6510LV; JEOL Ltd., Akishima, Tokyo, Japan). The specimens underwent dehydration in a desiccation chamber (K850 Semi-automated Critical Point Dryer; Quorum Technologies Ltd., Laughton, East Sussex, United Kingdom), were subjected to gold sputter-coating (JFC-1200), and were examined using SEM at 1,000x magnification at an acceleration voltage of 15 kV to confirm the mode of failure.

Data collection

The average μTBS values obtained from all beams from each tooth were then used for statistical analysis [15]. Failure modes were evaluated descriptively. The recorded numbers for each mode were calculated across all fractured beams within each group and presented as percentages. SEM images were obtained for qualitative analysis.

Statistical analysis

All statistical procedures were performed using IBM SPSS Statistics for Windows, version 25.0 (IBM Corp., Armonk, New York, United States). The data were evaluated for normality using the Kolmogorov-Smirnov test. The μTBS data were assessed for normality and homogeneity of variances using the Kolmogorov-Smirnov and Levene tests, respectively. A two-way ANOVA was used to evaluate the effects of adhesive system, treatment protocol, and their interactions on μTBS. Because the μTBS data deviated from normality and homogeneity of variance, differences among the six adhesive system x treatment protocol groups were evaluated using the Kruskal-Wallis test, followed by Dunn's post hoc test with Bonferroni adjustment. For all analyses, statistical significance was set at p < 0.05.

## Results

μTBS

Significant main effects of adhesive system (F (1, 54) = 603.964, p < 0.001, partial η² = 0.606) and treatment protocol (F (2, 54) = 162.758, p < 0.001, partial η² = 0.453) were revealed on μTBS, along with a significant interaction between adhesive system and treatment protocol (F (2, 54) = 13.934, p < 0.001, partial η² = 0.066). The mean μTBS of DES-OFL was significantly lower than both control-OFL and IES-OFL (all p < 0.001), and control-SBU was significantly higher than both IES-SBU and DES-SBU (all p < 0.001). DES-OFL and control-SBU, however, did not differ significantly (p = 1.000) (Table 4).

**Table 4:** Mean microtensile bond strength values for the adhesive and treatment protocol groups The different superscript capital letters indicate statistically significant differences, analyzed using Kruskal-Wallis followed by a Dunn’s post hoc test with Bonferroni adjustment (p < 0.05). IES: immediate endodontic sealing; DES: delayed endodontic sealing; OFL: OptiBond™ FL (Kerr Dental, California, United States); SBU: Single Bond Universal adhesive (Solventum Corporation, Eagan, Minnesota, United States); μTBS: microtensile bond strength

Treatment protocol	μTBS (MPa), mean ± SD	
	OFL	SBU
Control	57.59 ± 7.18^A^	41.12 ± 4.85^B^
IES	52.85 ± 7.22^A^	34.43 ± 5.97^C^
DES	40.98 ± 5.76^B^	30.27 ± 5.49^C^

Failure mode analysis

**Figure 2:**
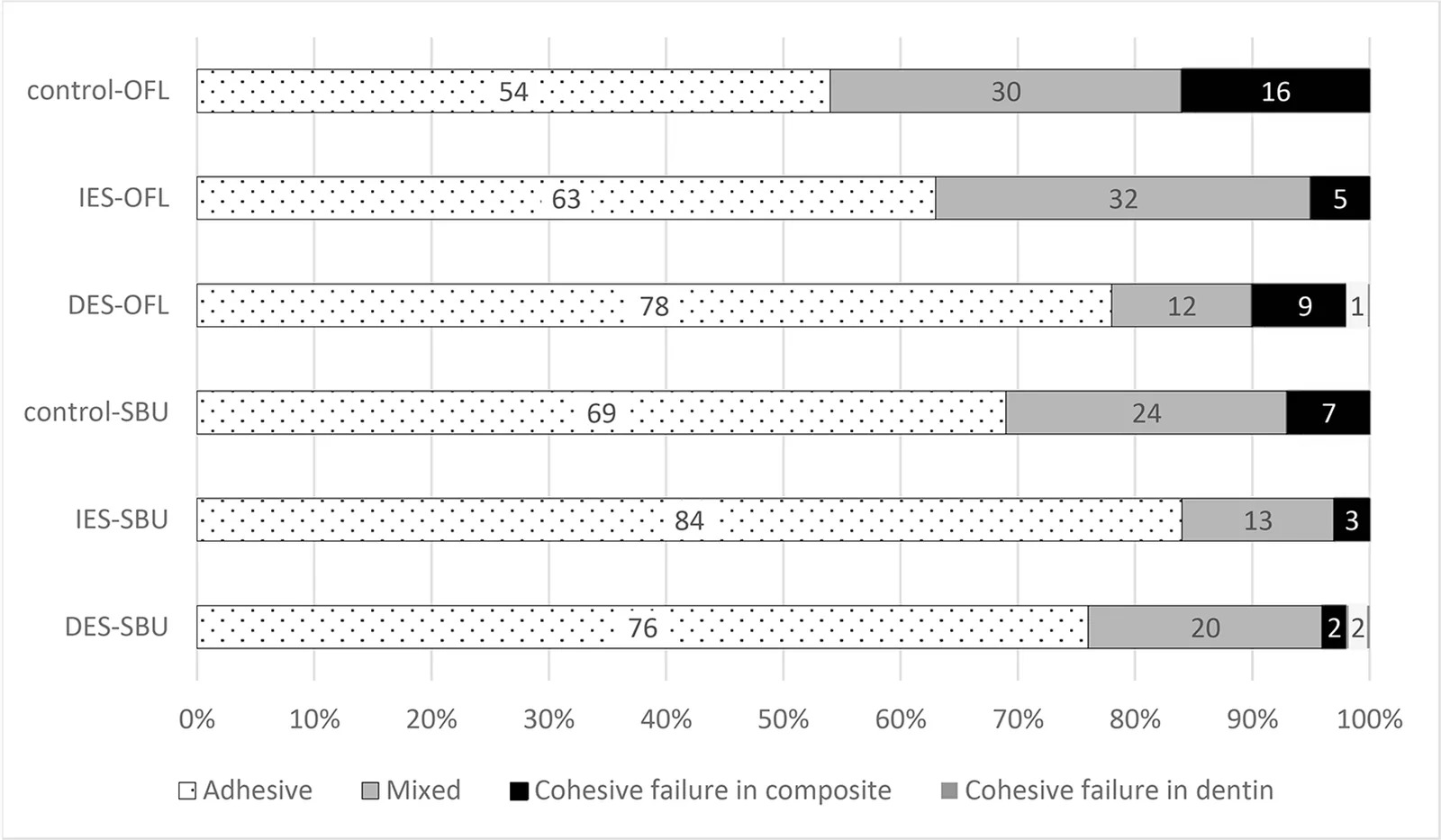
Percentage distribution of failure modes in each group Values within the bars indicate the percentage of specimens for each failure mode. IES: immediate endodontic sealing; DES: delayed endodontic sealing; OFL: OptiBond FL (Kerr Dental, California, United States); SBU: Single Bond Universal adhesive (Solventum Corporation, Eagan, Minnesota, United States)

**Figure 3:**
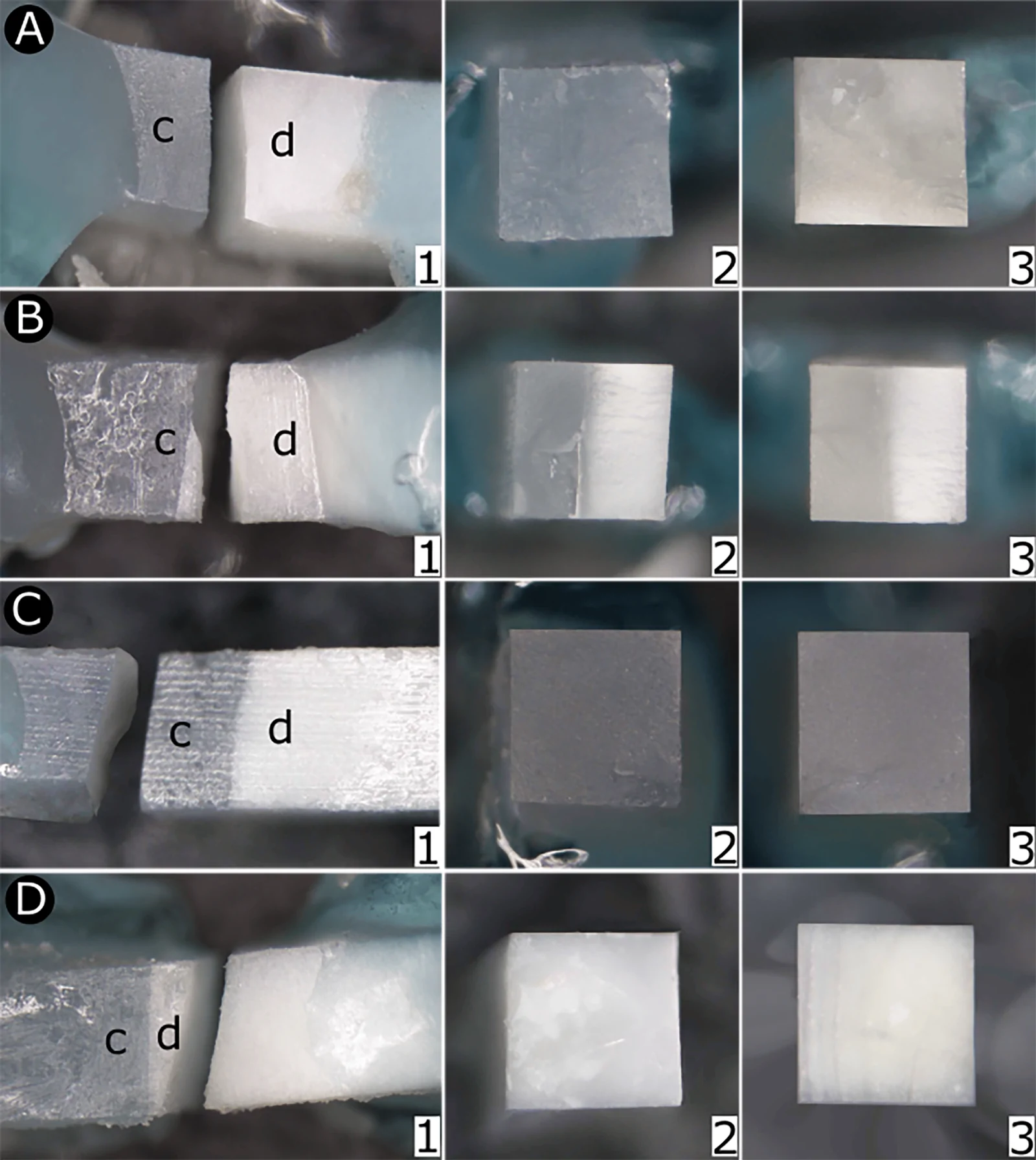
Representative stereomicroscope photographs of failure modes at 45x magnification of fractured specimens (1) Lateral view, (2) top view of the composite side, (3) top view of the dentin side. (A) Adhesive failure: Fractured surface of the composite side (c) is completely detached from the dentin side(d); (B) Mixed failure: Fractured surface of the composite side is partially detached from the dentin side; (C) Cohesive failure in composite: The lateral view of the fractured beam shows the fracture is in the composite side (c); (D) Cohesive failure in dentin: The lateral view of the fractured beam shows the fracture is in the dentin side (d).

**Figure 4:**
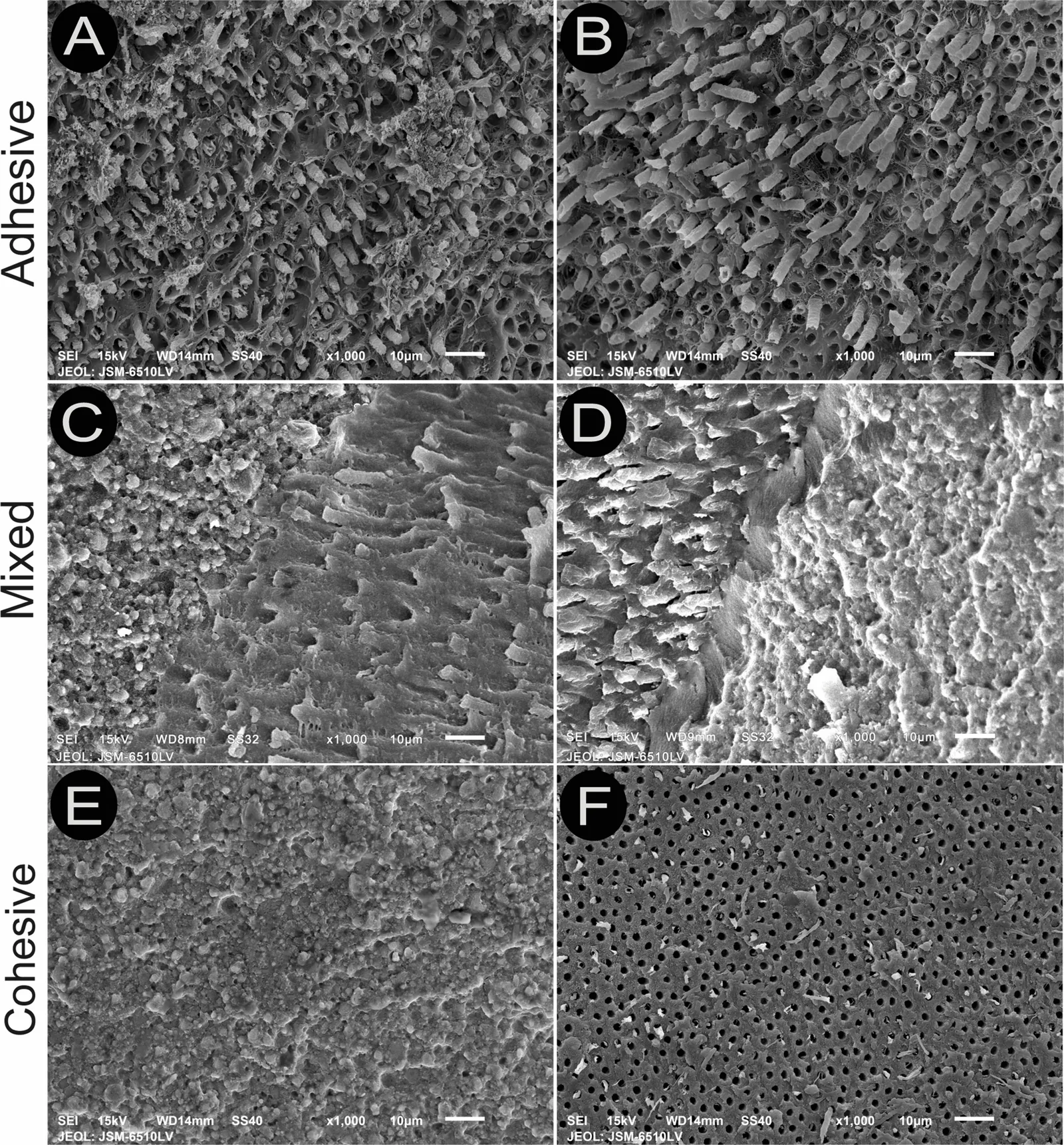
Representative SEM images of fractured surfaces of specimens at 1000x magnification depicting analysis of failure modes (A) Adhesive failure on the dentin side; (B) adhesive failure on the composite side; (C) mixed failure on the dentin side; (D) mixed failure on the composite side; (E) cohesive failure in composite; (F) cohesive failure in dentin SEM: scanning electron microscope

The distribution of failure modes, expressed as percentages for each group, is shown in Figure 2. The representative stereomicroscope photographs of the failure modes are presented in Figure 3. Representative SEM images of fractured surfaces for each failure mode are demonstrated in Figure 4.

Adhesive failure was the predominant failure mode in all groups, followed by mixed failure and cohesive failure in the resin composite. Cohesive failure in dentin was observed only in the DES treatment protocol, regardless of the adhesive system used.

## Discussion

Coronal sealability significantly influenced the success of endodontic treatment [16]. IES provides reliable bond performance for bonded restorations in the access cavities of ETT [17-19]. The present study investigated the impact of the IES treatment protocol on bond strength to intrapulpal dentin using different adhesive systems under a thermocycled aging process. The results revealed that the choice of adhesives impacted the efficacy of the IES treatment protocol, with multi-step adhesive systems demonstrating superior bond strength compared to simplified universal adhesives. Therefore, the study's null hypotheses were rejected.

**Figure 5:**
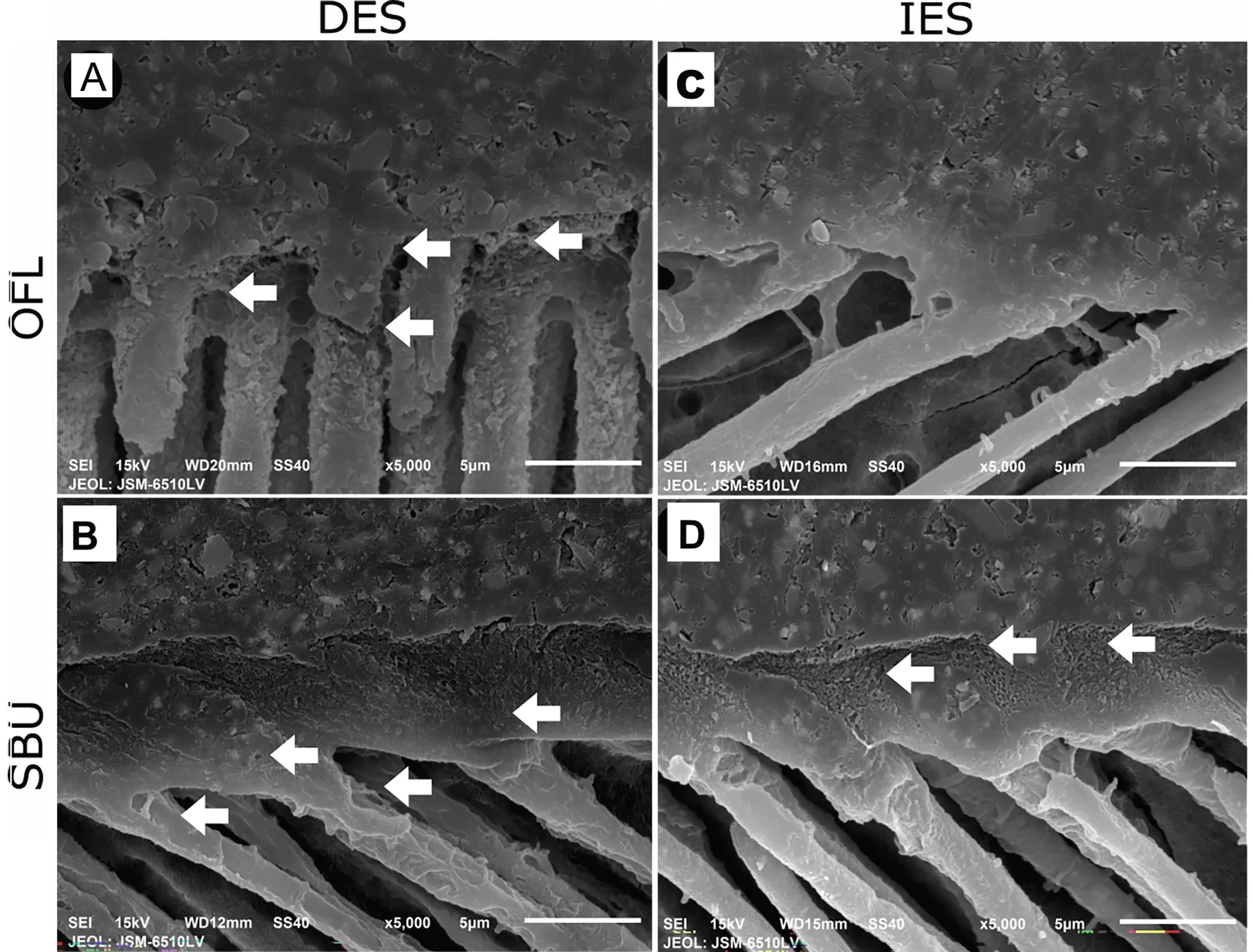
Representative SEM images of resin-dentin interfaces at 5000x magnification (A) DES-OFL group, (B) DES-SBU, (C) IES-OFL group, (D) IES-SBU group. The white arrows indicate clusters of voids. IES: immediate endodontic sealing; DES: delayed endodontic sealing; OFL: OptiBond FL (Kerr Dental, California, United States); SBU: Single Bond Universal adhesive (Solventum Corporation, Eagan, Minnesota, United States); SEM: scanning electron microscope

Dentin structure is well documented to be altered by NaOCl. This irrigant has been reported to degrade collagen [5] and alter the dentin matrix [13], thereby compromising the scaffold available for hybridization. Cohesive failure within dentin was observed only in the DES groups. This observation may be consistent with NaOCl-related alterations in dentin integrity, as previously suggested [20]; however, confirmation of the underlying mechanism should be done in future studies. In addition, residual oxidizing by-products of NaOCl are known to inhibit resin polymerization [6], further undermining the integrity of the resin-dentin interface. These NaOCl-induced alterations were particularly pronounced in the DES groups treated with either OFL or SBU, which exhibited significantly lower bond strengths relative to their respective control groups. This inhibitory effect was evident in Figures 5A, 5B (DES-OFL and DES-SBU, respectively); the hybrid and adhesive layers of both adhesive systems appeared irregular and contained numerous voids throughout the bonded interface. The findings may suggest a less uniform bonded interface in the DES groups.

The differences in bond strength between OFL and SBU can be explained by multifactorial reasons because these adhesive systems differ in several characteristics, including their hydrophobicity, filler content, functional monomers, solvent composition, and application protocols. Among these factors, differences in functional monomer chemistry may have contributed to their bonding behavior to intrapulpal dentin. The functional monomer used in OFL is glycerol phosphate dimethacrylate (GPDM), which was claimed to promote resin penetration into etched intrapulpal dentin [21]. In contrast, the chemical bonding contribution of SBU is mainly derived from 10-methacryloyloxydecyl dihydrogen phosphate (10-MDP), which ionically interacts with calcium in hydroxyapatite to form stable 10-MDP-Ca salts and nano-layering at the adhesive interface [22]. However, the use of SBU in the etch-and-rinse mode may further demineralize the dentin and reduce the hydroxyapatite available for chemical interaction. This effect may be more significant in pulp chamber dentin, where the reduced amount of intertubular dentin already limits the available mineralized substrate [23]. Therefore, differences in GPDM and 10-MDP chemistry may represent one of several factors associated with the different bond strengths observed between the control-OFL and control-SBU groups.

Another factor that may explain this difference is that the multi-step adhesive system (OFL) utilized a distinct hydrophobic resin that stabilized the hybrid layer and served as a stress-absorbing medium [10]. The adhesive layer acted as an effective protective barrier, as seen in the IES-OFL group in Figure 5C, shielding both the inorganic and organic components of the dentinal tissue from aggressive endodontic irrigants. This layer may prevent the percolation of harsh irrigants. Furthermore, the high filler loading within the hydrophobic resin acts as a stress-buffering barrier to counteract the polymerization shrinkage inherent in bonded restorations [24], as evidenced by the higher μTBS in both the control-OFL and IES-OFL groups. This high filler content also limits water sorption [25], allowing the interface to effectively withstand the challenging conditions imposed by both irrigants and artificial aging. Collectively, these factors ensure complete resin infiltration into the complex dentinal structure and the formation of a stable and durable resin-dentin bond interface.

In contrast to the multi-step adhesive, the universal adhesive (SBU) utilized in etch-and-rinse mode failed to provide a sufficient protective layer. The inferior bond strength of SBU may be attributed to its lower filler content and thinner adhesive layer. The thin layer may be insufficient to resist the polymerization stress [24] arising from the high configuration factor (C-factor) inherent in endodontic access cavities. Moreover, the permeable nature of the SBU adhesive layer, as indirectly shown in nanoleakage investigation [26], combined with the incorporation of both hydrophilic and hydrophobic monomers within a single layer, may allow the percolation of NaOCl. This facilitates the formation of voids and accelerates degradation, as seen in SEM observations in Figure 5D, which shows a cluster of voids in the adhesive layer, and reduced adhesive layer homogeneity in the IES-SBU group, thereby weakening the adhesive interface and reducing overall bond strength. To mitigate the inadequate protection provided by universal adhesives, the application of an extra-hydrophobic coating using a flowable resin composite has been recommended [24]. Such an additional layer reinforces the underlying thin adhesive and serves as a robust barrier against the percolation of aggressive endodontic irrigants. Furthermore, this layer may benefit subsequent restorations by facilitating stress-free bond development [27], thereby enhancing the long-term stability of the resin-dentin interface.

Compared with previous studies [18], the present study included several aspects intended to simulate clinical conditions. First, bonding performance was evaluated on the pulpal wall dentin, rather than on coronal dentin, which represented the substrate in the clinical situation. Compared with coronal dentin, pulp chamber wall dentin exhibits greater dentinal tubule density and larger tubule diameters and consequently contains less intertubular and peritubular dentin [23]. Differences in dentin substrate characteristics have been shown to influence bond strength outcomes [28]. In addition, previous studies used only 5 mL of 2.5% NaOCl [19], representing a relatively limited NaOCl exposure. Previous evidence has shown that 5.25% NaOCl applied for 10 minutes provides sufficient penetration into root canal dentin [29]. Moreover, a flow rate of 4 mL/min has been recommended to minimize apical extrusion [14]. Therefore, a total volume of 40 mL of 5.25% NaOCl was used in the present study to approximate a clinically relevant NaOCl exposure protocol on the dentin substrate under standardized conditions. The irrigation protocol was limited to NaOCl because we would like to isolate its influence on dentin bonding and reduce potential confounding effects from additional irrigants. Previous studies have reported comparable bond strength following NaOCl alone and NaOCl followed by ethylenediaminetetraacetic acid (EDTA) [18,30]. Nevertheless, the effect of combined NaOCl and EDTA irrigation, as a recommended clinical procedure, should be further investigated.

Clinical implications

From a clinical perspective, the development of modern endodontic instruments has facilitated more conservative access cavity preparation without compromising treatment outcomes, expanding the clinical application of direct composite restorations in ETT, particularly when substantial tooth structure remains. In this clinical scenario, preserving the integrity of the bonding substrate is essential. Clinical application of this protocol requires complete removal of caries and defective restorations, together with accurate identification of all root canal orifices, to ensure effective sealing of the access cavity. In line with a previous study [17], the present findings suggest that building up the temporary wall before endodontic treatment may help preserve bond strength to the dentin substrate. However, this potential benefit requires clinical validation.

Limitations

Interpretation of the present findings should be done with caution because of some limitations. The use of young, extracted third molars may not fully represent the dentin substrate of older or restored teeth encountered clinically. Currently, various commercial universal adhesive systems are available, which differ in composition and probably lead to different findings. Moreover, only the etch-and-rinse mode was conducted, suggesting further investigation of other clinical strategies of adhesive systems. Other artificial aging protocols such as mechanical loading or water storage are also suggested to expand the aging factor occurring under clinical conditions. Future investigations may also explore the chemical and molecular interactions underlying adhesive bonding to dentin. Additionally, allocation concealment from the operator performing specimen preparation was not formally implemented, which represents a potential source of bias despite the randomized, stratified design.

## Conclusions

IES preserved bond strength when performed with a filled, multi-step etch-and-rinse adhesive, producing resin-dentin interfaces comparable to untreated dentin. By sealing dentin before exposure to endodontic irrigants, this approach better preserved substrate integrity and bonding performance than DES. Within the limitations of the present study, IES with OFL resulted in higher bond strength than DES. Further clinical studies are required to validate these findings before this approach can be recommended for clinical use.

## References

[1] Gillen BM, Looney SW, Gu LS (2011). Impact of the quality of coronal restoration versus the quality of root canal fillings on success of root canal treatment: a systematic review and meta-analysis. J Endod.

[2] Sagsen B, Aslan B (2006). Effect of bonded restorations on the fracture resistance of root filled teeth. Int Endod J.

[3] Abu-Awwad M, Halasa R, Haikal L, El-Ma'aita A, Hammad M, Petridis H (2025). Direct restorations versus full crowns in endodontically treated molar teeth: a three-year randomized clinical trial. J Dent.

[4] Mario D, Mario A, Allegra C (2022). The influence of indirect bonded restorations on clinical prognosis of endodontically treated teeth: a systematic review and meta-analysis. Dent Mater.

[5] Perdigão J, Lopes M, Geraldeli S, Lopes GC, García-Godoy F (2000). Effect of a sodium hypochlorite gel on dentin bonding. Dent Mater.

[6] Lai SC, Mak YF, Cheung GS (2001). Reversal of compromised bonding to oxidized etched dentin. J Dent Res.

[7] Magne P (2005). Immediate dentin sealing: a fundamental procedure for indirect bonded restorations. J Esthet Restor Dent.

[8] De Rose L, Krejci I, Bortolotto T (2015). Immediate endodontic access cavity sealing: fundamentals of a new restorative technique. Odontology.

[9] Ahmad J, Sajjan GS, Panithini DB, Varma M, Satish RK, Prasad KD (2023). Effect of delayed endodontic sealing with dentin biomodification on fracture resistance of endodontically treated teeth. J Adv Oral Res.

[10] Magne P, Kim TH, Cascione D, Donovan TE (2005). Immediate dentin sealing improves bond strength of indirect restorations. J Prosthet Dent.

[11] Abo-Alazm EA, Safy RK (2022). Impact of immediate dentin sealing using universal adhesive under simulated pulp pressure on microtensile bond strength of indirect resin composite restorations and dentin permeability. Eur J Dent.

[12] Jafarnia S, Zeinaddini Meymand J, Zandkarimi F (2022). Comparative evaluation of microtensile bond strength of three adhesive systems. Front Dent.

[13] Vongphan N, Senawongse P, Somsiri W, Harnirattisai C (2005). Effects of sodium ascorbate on microtensile bond strength of total-etching adhesive system to NaOCl treated dentine. J Dent.

[14] Park E, Shen Y, Khakpour M, Haapasalo M (2013). Apical pressure and extent of irrigant flow beyond the needle tip during positive-pressure irrigation in an in vitro root canal model. J Endod.

[15] Sano H, Shono T, Sonoda H, Takatsu T, Ciucchi B, Carvalho R, Pashley DH (1994). Relationship between surface area for adhesion and tensile bond strength — evaluation of a micro-tensile bond test. Dent Mater.

[16] Ray HA, Trope M (1995). Periapical status of endodontically treated teeth in relation to the technical quality of the root filling and the coronal restoration. Int Endod J.

[17] Carvalho MA, Lazari-Carvalho PC, Maffra PE, Izelli TF, Gresnigt M, Estrela C, Magne P (2025). Immediate pre-endodontic dentin sealing (IPDS) improves resin-dentin bond strength. J Esthet Restor Dent.

[18] Marques JA, Falacho RI, Almeida G (2025). Advancing adhesive strategies for endodontically treated teeth-part II: dentin sealing before irrigation increases long-term microtensile bond strength to coronal dentin. J Esthet Restor Dent.

[19] Al-Rubaie AI, Al-Shamma AM (2025). Effect of immediate pre-endodontic dentin sealing on the cuspal deflection and fracture strength of endodontically treated teeth. Clin Oral Investig.

[20] Bronzato JD, Correa ACP, Gomes BP, Ferraz CC (2021). Residual effect of sodium hypochlorite on pulp chamber dentin adhesion. G Ital Endod.

[21] Yoshihara K, Nagaoka N, Hayakawa S, Okihara T, Yoshida Y, Van Meerbeek B (2018). Chemical interaction of glycero-phosphate dimethacrylate (GPDM) with hydroxyapatite and dentin. Dent Mater.

[22] Yoshihara K, Nagaoka N, Yoshida Y, Van Meerbeek B, Hayakawa S (2019). Atomic level observation and structural analysis of phosphoric-acid ester interaction at dentin. Acta Biomater.

[23] Fosse G, Saele PK, Eide R (1992). Numerical density and distributional pattern of dentin tubules. Acta Odontol Scand.

[24] de Carvalho MA, Lazari-Carvalho PC, Polonial IF, de Souza JB, Magne P (2021). Significance of immediate dentin sealing and flowable resin coating reinforcement for unfilled/lightly filled adhesive systems. J Esthet Restor Dent.

[25] Tang C, Mercelis B, Zhang F (2024). Filler mixed into adhesives does not necessarily improve their mechanical properties. Oper Dent.

[26] Ali RH, Niazy MA, Naguib EA, Abdel Ghany OS (2018). Effect of application technique and mode of curing on nano-leakage of universal adhesive system. Future Dent J.

[27] Reis A, de Oliveira Bauer JR, Loguercio AD (2004). Influence of crosshead speed on resin-dentin microtensile bond strength. J Adhes Dent.

[28] Toba S, Veerapravati W, Shimada Y, Nikaido T, Tagami J (2003). Micro-shear bond strengths of adhesive resins to coronal dentin versus the floor of the pulp chamber. Am J Dent.

[29] Zhang K, Tay FR, Kim YK (2010). The effect of initial irrigation with two different sodium hypochlorite concentrations on the erosion of instrumented radicular dentin. Dent Mater.

[30] Par M, Steffen T, Dogan S, Walser N, Tauböck TT (2024). Effect of sodium hypochlorite, ethylenediaminetetraacetic acid, and dual-rinse irrigation on dentin adhesion using an etch-and-rinse or self-etch approach. Sci Rep.

