# Correction: Comparative Analysis of Bond Strength in Endodontic Access: Immediate Endodontic Sealing Versus Delayed Endodontic Sealing

**DOI:** 10.7759/cureus.c481

**Published:** 2026-09-18

**Authors:** Thanyaporn Buranakarn, Yanee Tantilertanant, Junji Tagami, Somsinee Pimkhaokham

**Affiliations:** 1 Department of Operative Dentistry, Faculty of Dentistry, Chulalongkorn University, Bangkok, THA; 2 Department of Cardiology and Operative Dentistry, Graduate School of Medical and Dental sciences, Institute of Science Tokyo, Tokyo, JPN

The affiliations for Junji Tagami have been changed to: ‘Department of Operative Dentistry, Faculty of Dentistry, Chulalongkorn University, Bangkok, Thailand’ and ‘Department of Cardiology and Operative Dentistry, Graduate School of Medical and Dental sciences, Institute of Science Tokyo, Tokyo, Japan’.

